# Simulation-Based Analysis of the Effect of Alpha Irradiation on GaN Particle Detectors

**DOI:** 10.3390/mi14101872

**Published:** 2023-09-29

**Authors:** Jianming Lei, Nan Wang, Rukai Jiang, Qianyu Hou

**Affiliations:** 1 School of Electrical Engineering, Nanjing Vocational University of Industry Technology, Nanjing 210023, China; jmlei82@niit.edu.cn; 2 Research Center for Optoelectronic Materials and Devices, School of Physical Sciences and Technology, Guangxi University, Nanning 530004, China

**Keywords:** GaN, simulation, radiation detector, alpha irradiation

## Abstract

Radiation-hardened semiconductor GaN has drawn considerable attention owing to its excellent properties such as large displacement energy. Many studies have focused on evaluating the degradation of GaN-based power device performance by proton beam or particle irradiation, while quantitative analysis of the energy transfer process of particles inside the material and the mechanisms involved in inducing degradation of electrical properties are rare. Here, on the basis of the fabricated alpha-particle detector, a device model validated by basic electrical experiments is established to simulate the influence of alpha-particle irradiation on the leakage current of the device. We observe that the current does not change significantly with increasing radiation fluence at low bias, while it shows a descending trend with increasing radiation fluence at higher bias. However, increasing the energy of the radiation particles at the same radiation fluence directly leads to a monotonically elevated leakage current. Such a series of phenomena is associated with radiation-induced changes in the density of trapped states within the active layers of the device.

## 1. Introduction

In recent years, radiation detectors based on wide-bandgap semiconductors such as GaN have received increasing attention owing to their widespread applications in medical imaging, nuclear reactors, nondestructive inspection, and outer space [1,2,3,4,5]. Compared with the conventional semiconductor Si, which is limited in extreme environments, the radiation-hardened semiconductor GaN has been developed as a particle detector thanks to its wide bandgap energy and high thermal stability [6,7,8,9,10]. Compared with GaAs and diamond, GaN is also a competitive radiation-hardened material due to its large displacement energy of 50 eV (14 eV for GaAs and 43 eV for diamond) [11,12]. Many research groups have reported GaN alpha-particle detectors with different types of structures, some of which have successfully achieved a high charge collection efficiency (CCE) of approximately 100% at high voltage [13,14,15,16,17,18]. Meanwhile, to obtain a better understanding of the charge collection process inside the detectors, some researchers discussed the transient current by conducting numerical simulations [14,19]. Despite this progress, more systematic research is needed on the energy transfer mechanism of alpha particles within the GaN detectors. Quantitative analysis of the energy loss of incident alpha particles and recoil nuclei is essential to study the irradiation tolerance of devices.

Meanwhile, outer space or nuclear reactor applications put forward high requirements for the reliability of particle detectors for extended periods in extreme environments with high amounts of radiation. It is significant to evaluate the radiation resistance of particle detectors. Recently, many researchers have discussed the irradiation tolerance of GaN-based power devices by measuring the total ionizing dose response under different radiation sources, such as γ-rays, protons, and alpha particles [20,21,22,23]. However, a detailed exploration of how alpha-particle radiation affects the electronic properties of GaN radiation detectors is seldom reported.

In this work, the mechanisms concerning the electrical performance degradation of a GaN alpha-particle detector were systematically studied. We calculated the linear energy transfer (LET) and non-ionizing energy loss (NIEL) of incident alpha particles and recoil nuclei using the Monte Carlo program the Stopping and Range of Ions in Matter (SRIM) [24,25,26,27]. Subsequently, we established a technology computer-aided design (TCAD) device model, which was validated by applying the experimental data acquired from a fabricated GaN alpha-particle detector. Based on this model, the response of a GaN alpha-particle detector exposed to alpha radiation with different fluences and energies was discussed.

## 2. Device Fabrication and Model

**Figure 1:**
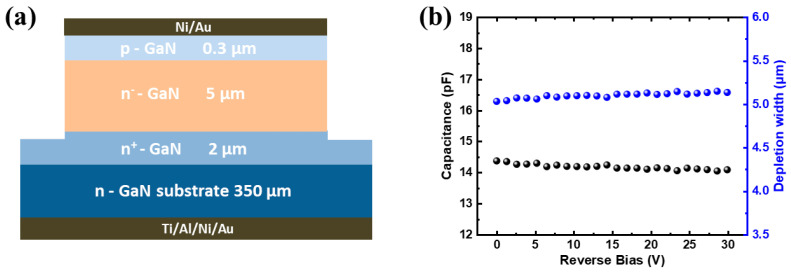
(**a**) Cross-sectional schematic diagram of the GaN alpha-particle detector. (**b**) Variations of the capacitance and depletion width of the detector as functions of reverse bias.

The GaN p-i-n structure in this work was grown on the 350 μm thick double-polished GaN substrate by metal-organic chemical vapor deposition. As shown in Figure 1a, the structure consists of a 2 μm thick n^+^-type GaN layer (n = 1 × 10^18^ cm^−3^), a 5 μm thick compensated n^−^-type GaN layer (n = 5.5 × 10^14^ cm^−3^), and a 0.3 μm thick p-type GaN layer (*p* = 1 × 10^18^ cm^−3^). Prior to the device fabrication, the wafer was cleaned with acetone, ethanol, and deionized water. After the pretreatment of photolithography, the fabrication process was started with mesa etching through the system of BCl_3_/Cl_2_-based inductively coupled plasma. The mesa with a diameter of 1 mm was etched to the n^+^-type GaN layer using SiO_2_ as an etching mask. To suppress the leakage current along the mesa sidewall, the wafer was immediately immersed in 0.1 mol/L KOH solution at 85 °C for 10 min [28]. Finally, the electrode metallization is deposited and patterned on both wafer sides to enable contact by ultrasonic wire bonding. Before measuring the energy spectra, the radioactive source and the packaged detector were placed in a shielded aluminum cylinder for electromagnetic shielding. Figure 1b shows the variations of the capacitance and depletion width of the detector as functions of reverse bias. There is a slight change in the value of capacitance for the reverse bias from 0 V to 30 V. The value of capacitance at 0 V is 14.3 pF. Therefore, the depletion width is calculated to be 5.03 μm, which indicates that the detecting layer of the detector can be completely depleted by the built-in electric field of the p-n junction without applied bias voltage due to the low carrier concentration of the detecting layer.

**Figure 2:**
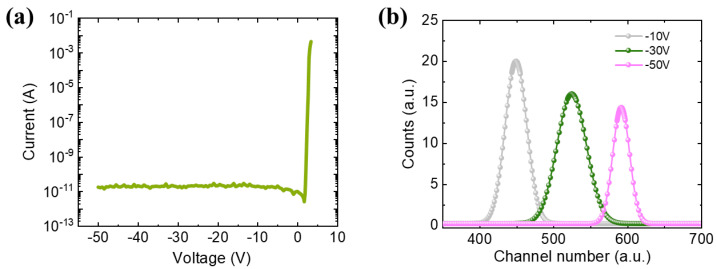
(**a**) I-V characteristic of the GaN alpha-particle detector. (**b**) The measured ^241^Am alpha particle spectra obtained from the GaN alpha-particle detector with different reverse biases.

Figure 2a shows the current-voltage (I-V) characteristic of the fabricated p-i-n GaN alpha-particle detector. The leakage current is 7 pA at −1 V and maintains a low value below 30 pA at −50 V. Figure 2b shows the measured ^241^Am alpha particle spectra obtained from the GaN alpha-particle detector under different biases. The electric field intensity ascends continuously as the voltage increases, which is beneficial to reduce the recombination probability of the generated electron-hole pairs. This increases the collected charge, causing the peak centroid of the energy spectrum to shift in the positive direction. At the reverse bias of 50 V, the peak centroid is 590 channels, corresponding to a collected charge of 1.44 MeV. These experimental results were used to verify the accuracy of the developed simulation model later.

We used the SRIM based on Coulombic and nonrelativistic interactions to analyze the stopping power of alpha particles in GaN and calculate the values of LET and NIEL. Moreover, a TCAD based on the ATLAS device simulator was employed to investigate the degradation of the electrical properties of the GaN alpha-particle detector. Poisson’s equation and continuity equation were solved in the simulation. For carrier mobility, the concentration-dependent mobility model and the lateral electric field-dependent mobility model were included. The Shockley-Read-Hall recombination and Auger recombination models were set for carrier recombination. In addition, the radiation fluence model was employed to simulate the defect dislocation generation rate due to alpha particle bombardment in GaN. Table 1 shows the key parameters used in the simulation.

## 3. Results and Discussions

Table 2 shows the calculated energy loss in GaN for the alpha particles with an energy of 5.486 MeV using SRIM. It should be noted that the stopping mechanism of alpha particles in GaN is dominated by ionizing energy loss, accounting for 99.70%. Moreover, it is worth noting that the energy of recoil nuclei is mainly lost by phonons. Moreover, the alpha particles also transfer part of their energy to the target atoms due to the collision. After the collision, if the energy of the target atoms is less than the atomic displacement energy, the target atom will return to the original lattice position, resulting in energy loss through the phonon mode. Only if the energy of the target atom is greater than the displacement energy, the atoms will be knocked away from the lattice position, eventually generating a vacancy. When the energy of the target atom is large enough, cascade collisions will occur, generating more vacancies.

**Figure 3:**
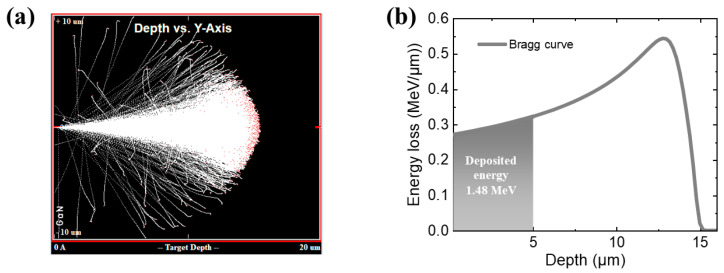
(**a**) Monte Carlo simulation of alpha particle bombardment of the GaN layer using TRIM. (**b**) Bragg ionization curve of 5.486 MeV alpha particles in the GaN layer.

To obtain a better understanding of the collision between alpha particles and GaN, Figure 3a shows the Monte Carlo simulation of alpha-particle (~5.486 MeV) bombardment of the GaN layer using Transport of Ions in Matter (TRIM) simulation. From the figure, it is observed that the range of alpha particles with an energy of 5.48 MeV in the GaN layer is approximately 15 µm. This indicates that the thickness of the GaN absorption layer (~5 μm) is not enough for the full energy deposition. SRIM simulation can be used to determine the relationship between energy deposition and the penetration depth of 5.48 MeV incident alpha particles in GaN. As shown in Figure 3b, the Bragg ionization curve shows that a total energy loss of 1.48 MeV is deposited within the 5 µm GaN layer.

**Figure 4:**
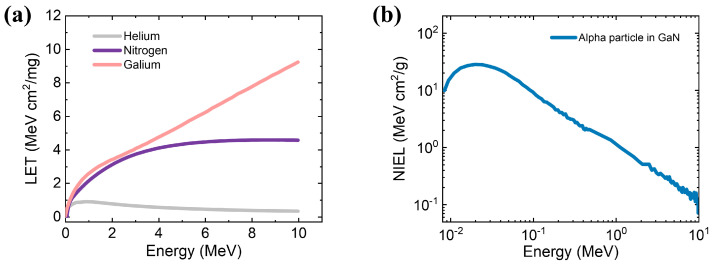
(**a**) LET of ions in GaN as a function of ion energy. (**b**) NIEL of alpha particles in GaN as a function of ion energy.

Commonly, the energy loss to charge ionization is quantified by LET. Figure 4a shows the LET of ions in GaN as a function of ion energy using SRIM. As the atomic number increases, the value of LET keeps going up, which is similar to the simulated results (proton) of ref. [29]. The LET of Ga recoil atoms is monotonically increasing in the observed energy range. However, the LET curves of He and N recoil ions are likely to show a trend of rising first and then declining slowly with the increase in ion energy. To analyze the transient response of the device by simulation, the linear charge deposition (LCD), which defines the actual charge deposited in units of pC/μm, is calculated. In GaN, it is considered that about 8.9 eV is required to generate an electron-hole pair [30]. Thus, the conversion factor from the value of LET to LCD is about 0.004. When the energy of alpha particles is 5.486 MeV, the value of LCD is calculated to be 0.002 pC/μm.

Another energy loss mechanism is the non-ionizing process quantified by NIEL, which depends on the target material and incident particles. It is worth mentioning that plenty of studies have successfully demonstrated that the degradation of semiconductor devices after irradiation is greatly related to NIEL [31,32,33]. Analysis of NIEL helps predict the radiation resistance limitation of GaN devices. Figure 4b shows the calculated NIEL of alpha particles in GaN as a function of energy. As can be seen, NIEL exhibits a decreasing trend with increasing energy. This trend can be explained by the higher energy with fewer interactions during the collision. When the energy of alpha particles is 5.486 MeV, the NIEL is about 0.21 MeV cm^2^/g. This value is close to the result of large-scale molecular dynamics simulations [11].

**Figure 5:**
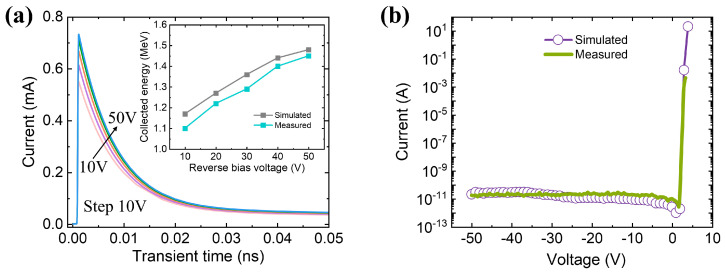
(**a**) The transient current of the GaN alpha-particle detector under different reverse biases induced by a 5.486 MeV alpha particle. The inset is the comparison of the simulated and measured collected energies. (**b**) The simulated and measured I-V characteristics of the GaN alpha-particle detector.

To analyze the charge collection process and validate the established model, we simulate the transient current with different bias voltages of the GaN alpha-particle detector using TCAD. The structure parameters of the detector are consistent with those of the above experiment as shown in Figure 1a. The simulation model assumes that an alpha particle with an energy of 5.486 MeV is incident perpendicular to the detector surface. The resulting electron-hole pair charge cloud is about 5 μm in length. As shown in Figure 5a, the charge collection time is about 1 ns. Furthermore, the current peak gradually rises with the increasing bias voltage. As is well known, the collected charges are the integration of time-dependent current curves. The inset of Figure 4a shows the corresponding simulated and measured collected energy. As can be seen from the figure, there is a steady upward trend of the simulated and measured collected energy with the increasing bias voltage. Meanwhile, the simulated energy is slightly higher than the measured ones. The explanation for the result is that the trapping centers introduced by the growth and fabrication process lead to the incomplete collection of charge of the fabricated detector. In addition, Figure 5b provides the simulated and measured I-V characteristics of the GaN alpha-particle detector. The simulated and measured I-V characteristics are highly fitted in the figure. These results obtained from the comparison of the collected energy and I-V curves strongly validate the established device model.

**Figure 6:**
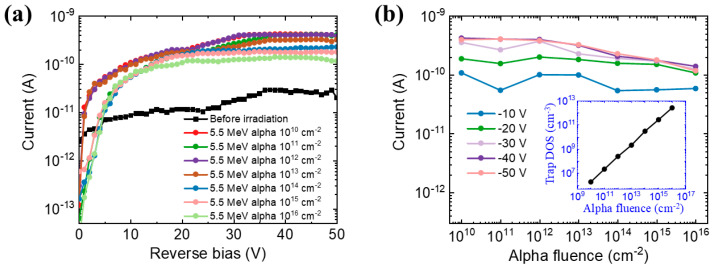
(**a**) The I-V characteristics of the GaN alpha-particle detector under alpha irradiation at various fluences from 10^10^ to 10^16^ cm^−2^. The alpha energy is 5.5 MeV. (**b**) The current as a function of the alpha fluence under different bias voltages from −10 V to −50 V. The inset shows the dependence of the trap DOS within the active layers of the device on the alpha fluence at −50 V.

Based on the validated device model and NIEL values, we simulated the I-V characteristics of the detector before and after alpha irradiation using TCAD. Figure 6a shows the I-V characteristics of the device exposed to alpha radiation with sequentially increasing fluences from 10^10^ to 10^16^ cm^−2^. The energy of alpha particles is 5.5 MeV. Before the irradiation, the reverse leakage current rises slowly with the increasing voltage and reaches about 2 × 10^−11^ A at −50 V. Upon the irradiation, these current curves have similar shapes. At lower reverse bias, the current curves show an upward trend with increasing voltage, which is highly consistent with the experimental results of ref. [18]. As the reverse bias voltage increases, the current gradually approaches saturation. Furthermore, the post-irradiation current increases by about an order of magnitude relative to the pre-irradiation one. At the reverse bias of 50 V, the current increases to 3 × 10^−10^ A with a radiation fluence of 10^10^ cm^−2^. The difference in the current before and after irradiation can be explained by the explosion of electron-hole pairs due to alpha particle bombardment. In addition, the curves show that the current gradually declines as the fluence increases. This trend implies the incomplete collection of the generated electron-hole pairs in the device, which is caused by displacement damage due to irradiation.

It has been reported that displacement damage in semiconductors generally leads to the degradation of carrier mobility and lifetime, as well as carrier removal [34,35]. To obtain a better insight into the current degradation behavior, the current as a function of the alpha fluence under different bias voltages is shown in Figure 6b. We observe that the current does not change significantly with increasing radiation fluence at low bias. At the higher reverse bias of 50 V, the current decreases by 70% with the fluence rising from 10^10^ to 10^16^ cm^−2^, which indicates the strengthening of the displacement damage caused by irradiation. To clarify this point more clearly and evaluate the radiation hardness of the GaN device, we derived the radiation-induced trap density of states (DOS) within the active layers of the device under different fluences as shown in the inset of Figure 6b. The post-irradiation current of GaN detectors is strongly dependent on the carrier lifetime and mobility. With the rise of the alpha radiation fluence, the number of displacement defects would increase. These defects act as the scattering and trapping centers, leading to the scattering and trapping of the generated carriers. Thereby, the contribution of the generated electron-hole pairs to the current is reduced with the increase in alpha fluence.

**Figure 7:**
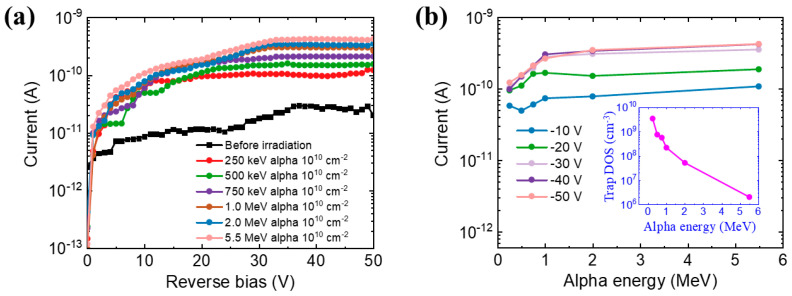
(**a**) The I-V characteristics of the GaN alpha-particle detector under alpha irradiation with different alpha energies from 0.25 to 5.5 MeV. The fluence is 10^10^ cm^−2^. (**b**) The current as a function of the alpha energy under different bias voltages from −10 V to −50 V. The inset shows the dependence of the trap DOS within the active layers of the device on the alpha energy at −50 V.

Moreover, we also investigate the effects of alpha irradiation with different energies from 0.25 to 5.5 MeV on the I-V characteristics of the device. The fluence of the alpha particles is 10^10^ cm^−2^. As shown in Figure 7a, there is a general rising trend of the current with the increasing alpha particle energy. More details are shown in Figure 7b. The current has increased nearly three times with the alpha energy increasing from 0.25 to 5.5 MeV at the reverse bias of 50 V. As mentioned above, the higher energy corresponds to the higher LET value, which means generating more electron-hole pairs. The NIEL value drops off with the increase in the energy of alpha particles, which indicates less displacement damage with higher energy alpha irradiation. Moreover, these results are consistently proved by the decreasing number of radiation-induced traps with ascending alpha particle energy, as displayed in the inset of Figure 7b.

## 4. Conclusions

In summary, this project was undertaken to evaluate the effects of alpha irradiation on the GaN alpha-particle detector. We investigated the energy loss process of low energy (<10 MeV) alpha particles in GaN based on the Coulombic and nonrelativistic interaction, and calculated the values of LET and NIEL in GaN. Moreover, a TCAD device model was established to evaluate the radiation displacement damage in devices, which is validated by a fabricated GaN alpha-particle detector. Based on the simulation results, it was concluded that with exposure to alpha irradiation, the reverse leakage current descended with the increase in alpha fluence and ascended with the increase in alpha energy. In addition, this study provides the first comprehensive assessment of the alpha irradiation hardness of GaN particle detectors. Although a methodology for the theoretical study of irradiation damage is discussed, which may be promising to generalize to other particle irradiation and semiconductors, considerably more experimental work will need to be conducted to determine the displacement damage caused by the alpha particles.

## Figures and Tables

**Table 1:** The key parameters in simulation.

Parameters	Values
Density of states N_c_ (cm^−3^)	2.24 × 10^18^
Density of states N_v_ (cm^−3^)	2.51 × 10^19^
Intrinsic carrier concentration n_i_ (cm^−3^)	1.06 × 10^−10^
Lifetime (electron)	1.0 × 10^−9^
Lifetime (hole)	1.0 × 10^−9^
Effective mass (electron)	0.2
Effective mass (hole)	1.0
Saturation velocity (electron) (cm/s)	1.91 × 10^7^
Saturation velocity (hole) (cm/s)	1.0 × 10^6^

**Table 2:** Energy loss of alpha particles in GaN.

Energy Loss (%)	Ions	Recoils
Ionization	99.70	0.06
Vacancies	0.00	0.01
Phonons	0.03	0.20

## Data Availability

The data presented in this study are available on request from the corresponding author.
